# Why do women not adhere to advice on maternal referral in rural Tanzania? Narratives of women and their family members

**DOI:** 10.1080/16549716.2017.1364888

**Published:** 2017-08-31

**Authors:** Andrea B. Pembe, Columba K. Mbekenga, Pia Olsson, Elisabeth Darj

**Affiliations:** ^a^ Department of Obstetrics and Gynaecology, School of Medicine, Muhimbili University of Health and Allied Sciences, Dar es Salaam, Tanzania; ^b^ Department of Women’s and Children’s Health, International Maternal and Child Health (IMCH), Uppsala University, Uppsala, Sweden; ^c^ School of Nursing and Midwifery, Aga Khan University, Dar es Salaam, Tanzania; ^d^ Department of Public Health and Nursing, NTNU, Norwegian University of Science and Technology, Trondheim, Norway; ^e^ Department of Obstetrics and Gynaecology, St Olav’s Hospital, Trondheim, Norway

**Keywords:** Health systems, gender, community, developing countries, social capital, maternal referrals, rural

## Abstract

**Background**: In most low-income countries, many women with high-risk pregnancies and complications do not reach the referral hospitals despite the provision of referral advice.

**Objective**: To explore how antenatal maternal referral advice is understood and handled in a rural Tanzanian community.

**Methods**: Individual in-depth interviews were conducted with six women who did not go to hospital and 13 people who were involved in the referral advice. Narrative analysis was used to describe and create meanings out of the decision-making process.

**Results**: In all interviews, not following the referral advice was greatly influenced by close family members. Three main traits of how referral advice was understood emerged: convinced referral is not necessary, accepting referral advice but delayed by others, and passive and moving with the wind. The main reasons given for declining the referral advice included discrediting midwives’ advice, citing previous successful deliveries despite referral advice; being afraid of undergoing surgery; lack of support for care of siblings at home; and high costs incurred during referral.

**Conclusions**: Declining maternal referral advice centred around the pregnant women’s position and their dependence on the family members around them, with a decreased ability to show autonomy. If they were socially and economically empowered, women could positively influence decision making during maternal referrals.

## Background

Referral during pregnancy is essential to ensure that women with high-risk pregnancies and complications access immediate and appropriate care [1]. If referral during pregnancy is utilized appropriately by women, it is expected to decrease maternal and newborn morbidity and mortality [2]. In Tanzania, as in most low-income countries, women with high-risk pregnancy and complications are referred to higher level facilities in accordance with the guidelines [3]. Despite the provision of referral advice in the lower level facilities, a substantial number of women do not reach the referral hospitals [4–7] and only 64% are delivered by a skilled birth attendant [8]. The Three Delay Model by Thaddeus and Maine [9] illustrates studies investigating barriers to adherence to referral advice for hospital delivery in rural Tanzania, including financial constraints, difficulty in accessing transport, poor quality of care and shortage of staff [5,10–12]. However, another study in southern Tanzania reported that pregnant women do not accept referral because they perceive that they will be separated from their families and fear the unfamiliar environment of the hospital [13]. Therefore, there is a need to explore the complex influences in the social environment that may prevent women in rural Tanzania adhering to the referral advice given by health workers.

Social capital is defined as ‘the collective value of all social networks and the inclination that arises from these networks to do things for each other’ and is mostly regarded to have positive health consequences in terms of social connections which enable individuals to have access to resources [14]. The concept extends beyond the accessibility of resources to an individual’s identity, participation, reciprocity and trust within the group. Social capital is expected to be more positive in rural communities, which commonly have poor health systems and a scarcity of resources [15,16]; individuals therefore rely on social networks when faced with health or social problems. Restriction of individual freedom and downward levelling of norms have been noted to be the negative consequences of social capital [17]. Pregnant women referred to hospitals from rural areas can face both the positive and negative consequences of social capital, as going or not going for the referral may be dependent on the social norms and connections that they have with their families and the community. Connell’s relational theory of gender [18] delineates gender as a social structure with four dimensions – economic, power, affection and symbolic – which operates within and between individuals, institutional and societal levels.

To assess the roles and interaction of the pregnant women and other actors in the family when maternal referral is advised, we anticipated that a detailed study of stories told by different actors engaged in this decision process would generate useful and new information. These insights will give us insight into what maternal choices and decisions are based on. This will be important in helping stakeholders in the Tanzanian reproductive healthcare system to understand why their antenatal advice for referral to a higher health facility may not be followed, despite the link between women’s participation in antenatal care and high maternity mortality, often in combination with neonatal deaths.

This article aims to explore how antenatal maternal referral advice is understood and handled in a rural Tanzanian community, by childbearing women and other people identified as partaking in the decision to decline the advice. Specifically, we study their considerations and interactions related to the decision-making process.

## Methods

### Study design

A qualitative research design using individual in-depth interviews [19] was adopted as it gives detailed and complex insight into people’s thoughts on daily issues and decision making around adherence to maternal referral advice.

### Study setting

The study was conducted in the catchment population of one of the dispensaries in Rufiji district, Tanzania. The dispensary provides antenatal care to pregnant women with low-risk pregnancies and refers women with high-risk pregnancies or complications to the hospitals in the district. Normal deliveries are conducted at the dispensary, and when a complication occurs during or after delivery the woman is referred to one of the hospitals. Transport to the hospitals is the responsibility of the referred woman and her relatives. They have to identify and negotiate payment for the transport and arrange for a person to escort the woman to the hospital. Rarely, a car from the district hospital is used, but if so, the woman and/or her relatives have to contribute about 30 litres of diesel for the transportation. The Rufiji district has two hospitals, a faith-based hospital, where maternal services are provided at subsidized cost, and a government-owned district hospital, where the services are free. Both hospitals provide comprehensive emergency obstetric and neonatal care services. In the study district, there are five health centres and 52 dispensaries. The district is rural and majority of the population in the district are Muslims and depend on agriculture, growing rice, maize, sorghum and cassava. A few people depend on fishing and keeping livestock. Ndegereko people, Matumbi, Ngindo, Nyagatwa, Pogoro and Makonde are the main ethnic groups living in the district. Despite the presence of different ethnic groups, all people in the area speak Kiswahili, the national language. Details of the district are described elsewhere [6,11].

### Sampling of respondents

Purposive sampling [19] was used to select six women who were initially involved in a longitudinal study of maternal referrals in the district [6] and did not reach any of the hospitals after receiving referral advice. Their referral indications included having five or more pregnancies, being younger than 20 years of age, the presence of anaemia, malposition of the baby and previous caesarean section. Previous studies reported that the last two indications had high acceptance as reasons for maternal referral to hospital, while the first two indications had low acceptance [6,11].

### Data collection

In-depth interviews were conducted first with the women who did not attend the hospital after referral advice. During the interview with the women, other people involved in the process of referral were identified. Nineteen people were identified by the women as being involved in the referral advice. Six of these people were not available for the interview as some had moved out of the area and the others were working at a distance and could not be reached at the time of the interview. All 13 interviews were conducted at a time convenient for the interviewee, in a secluded place on the interviewee’s compound, away from other family and community members to maintain privacy.

The first (ABP) and the second (CKM) authors are both Tanzanians experienced in qualitative data collection. CKM, a female nurse, conducted interviews with all the women, and ABP, a male obstetrician, interviewed the men. This was done purposely to allow respondents to talk freely during the interview and to respect gender sensitivity. The interviews started by posing a question and a request to the interviewee, ‘When were you aware of the advice that you [in interviews with women] or that your partner/family member [in interviews with people involved in the decision not to follow the referral] were to be referred to hospital for giving birth/further assessment and what happened when the information was given? Tell me about what happened after you learned of the need for referral’. Probing questions were used to clarify reactions towards the referral advice, action taken and challenges experienced in relation to the people involved in the referral advice. At the end of each interview, the interviewees were given the opportunity to ask questions. After each interview, the interviewer took notes on general impressions, contextual matters and non-verbal communication observed. The interviews were tape-recorded with the permission of the interviewees. The recordings were transcribed in the original language, Kiswahili, and translated into English to make them accessible to the non-Kiswahili-speaking researchers. Transcripts were checked for accuracy against audio-recordings and minor corrections were made.

### Data analysis

Narrative analysis [20] was used to describe and create meanings out of the complex decision making on non-adherence to maternal referral advice captured in the interviews. The analysis allows for the exploration of human experiences and/or social phenomena through the form of the content of stories analysed as textual units. First, all interviews were read several times and organized in six cases centred on the interviews of each woman given the referral advice. Secondly, all interviews pertaining to each case were read in the light of the referred woman’s experiences. Thirdly, comparisons were made within and between the cases regarding the diverse understandings of the referral advice, as well as the considerations and interactions among all those engaged in the decision. Thereby, differences and similarities between the cases were identified. Fourthly, reconstruction of the stories [21] was undertaken by creating three narratives that captured the main traits of all cases, each with a clear beginning and ending.

## Results

All other people identified by the woman as being involved in the referral process were family members. The basic characteristics of the respondents, their relationships and the indications for referral are summarized in Table 1.

**Table 1.** Overview of interviewees’ characteristics and indications for referral.

Referred woman	Age (years)	Marital status	Gravidity^a^	Parity^a^	Education	Indication for referral^b^	Involved persons
M 1	18	Single	2	1	Primary education	Breech presentation	Mother, father, aunt, partner^c^
M 2	35	Married	10	9	Primary education	Grand multigravidae	Husband, aunt, sister^c^
M 3	16	Married	1	0	No formal education	< 20 years, anaemia	Mother, husband, aunt^c^
M 4	26	Married	3	1	Primary education	Severe anaemia	Mother, husband, sister-in-law^c^, father^c^
M 5	25	Married	3	2	No formal education	Previous caesarean section	Husband, brother-in-law^c^
M 6	30	Separated	3	2	No formal education	Previous caesarean section	Mother, father-in-law, stepfather

^a^Gravidity and parity at the time of referral advice.

^b^Indication for referral recorded in the register during the follow-up study.

^c^Not available for interview.

Common to all cases was the pregnant woman’s failure to follow the referral advice. The overall sequence of women’s ways of conveying the information about the referral advice given at the health facility was also similar in all cases. A woman usually informed her husband upon her return from the health facility. If he was not around, the woman discussed the referral with her mother or mother-in-law. Women living with their parents spoke first to their mothers and the information was then given to the woman’s father or father-in-law by the pregnant woman’s mother. Thereafter, family members including aunts, brothers-in law and sisters-in-law were informed about the advice given. Three narratives delineate the main traits of how referral advice is understood and handled. To preserve anonymity, all names in the narratives are fictitious.

### Narrative I: Jamila – convinced referral is not necessary

Jamila is 21 years old, married, and lives with her husband and their 2-year-old daughter in a village close to her parents’ village, which is 15 km away. When Jamila was 8 months pregnant, she was advised to go to the hospital for further assessment and delivery. The midwife had said that the baby’s buttocks were coming first. ‘In the first pregnancy, I attended antenatal care and had no problems during the birth at home. I was assisted by my mother, who is trained as a birth assistant and there was no problem’. When she returned from the dispensary, Jamila did not tell anyone about the advice during the second pregnancy, to give birth at the hospital. However, her husband came to know about the advice 3 days later when he found and read the antenatal card. ‘I told my husband that I will deliver at home irrespective of what is written in the card as going to the health facility is waste of time’. Jamila’s husband was worried that if a complication should occur it would be difficult to reach the dispensary, and was not sure that his wife was making the right decision. So he asked her to discuss it with her parents, as they had experience on these matters and would be contacted if a problem arose; and she followed his advice.

'My parents discussed and my mother supported me. She had seen women giving birth with the baby’s buttocks coming first at the dispensary without problems. Midwives do not always know, she says, sometimes they say the baby comes with buttocks first but during labour you find it presents by head.'

Jamila’s father, however, was worried that Jamila’s life and that of the baby would be in danger. He was undecided, but relied on Jamila’s mother’s decision.

Two days later, Jamila, her husband and parents met to discuss the situation. Her father asked whether Jamila’s husband had money in case an emergency referral was needed. Jamila’s husband said he had some money, but Jamila’s father said that it was not enough and more was needed. Jamila’s parents decided that Jamila would not go to the hospital but would deliver at the dispensary. ‘But I still wanted to deliver at home. I was worried about delivering at the nearby dispensary. The midwives had told me that, I would not be accepted because my baby had bad lie [breech]’. Jamila’s husband expressed his fear of losing Jamila and the baby, but he could not do anything except follow her parents’ decision.

Jamila, her husband and their first child were invited to move in with her parents, and said that they would go to the dispensary when labour started. ‘And we moved there, I was resting while my mother and other relatives were doing all the house chores and taking care of my first baby. My mother kept encouraging me that everything will be well’.

When labour started, her aunt was called to help take Jamila to the dispensary. Jamila’s mother asked the midwives to help Jamila deliver at the dispensary. The midwives told her mother that they would allow her to deliver, but informed the family that if anything bad happened it should be known that they had referred her to the hospital. Her mother said she had left everything to God. Labour took longer than expected, so Jamila’s father and husband were called. Jamila’s mother told Jamila’s father and husband to look for transport as Jamila may not be able to deliver at the dispensary. Jamila’s father asked Jamila’s aunt to make a call to Jamila’s uncle to help them get transport. Jamila’s uncle told them he would pay for the transport when he returned. Jamila’s father and husband went to look for transport, but before they returned, Jamila delivered her baby but with difficulty. ‘I told them that baby presenting with buttocks is a serious problem and what happened was God’s will’.

### Narrative II: Mariam – accepts referral advice but delayed by others

Mariam was 42 years old, married and in her seventh pregnancy. At her first antenatal care visit, she was asked to bring her husband next time for advice on family planning and to plan for the delivery.

'On that visit we were advised that I should go to deliver at the hospital because I had more than six deliveries. They told us to make preparations including saving money to be used for transport and living expenses while at the hospital, and also identify a person to stay with the other children at home.'

Once they returned home, her husband scorned the advice to go to the hospital for delivery, saying that she had previously delivered her babies without a problem. He further said that he did not have money for the referral; the money he had was for other family issues. ‘I tried to convince him of the importance of going to the hospital for delivery, but he was adamant and did not want to discuss the referral advice’. Mariam informed her younger sister, who discussed the referral advice with their aunt and the arguments Mariam had had with her husband. Mariam’s aunt, an experienced traditional birth attendant, lived in the same village. The aunt talked to Mariam and asked her what she wanted to do. ‘And I said that in the late months of the pregnancy, I wanted to go and stay with my husband’s brother’s wife, who lives close to the hospital, so that when labour starts I can easily reach the hospital’. The aunt dismissed the idea, saying that it was not easy as her husband’s brother was not there and Mariam was not directly related to her sister-in-law. If the husband were there it would be easier to arrange. The aunt advised Mariam not to work too much, and that the older children should help her with the household chores. ‘My aunt told me that we can go to hospital when a serious problem happens, as most people can help only when they have seen there is a serious problem’.

The aunt told Mariam that at the hospital they perform unnecessary operations to remove the baby, but Mariam’s sister told Mariam that they do not operate unnecessarily on pregnant mothers. The midwives and doctors at the hospital examined every woman and only those in need of an operation would be operated on, while others would be observed and then deliver normally. Neighbours had frightened Mariam by telling her that she might not wake up if she was operated on, but that if she did wake up she may suffer other long-term problems such as the sutured area getting tight during the cold season, not able to work well and having abdominal pain.

'I was afraid of the problems I heard of, and thought that if I was operated I will not be able to take care of my child and not be able to do house chores. I will have to depend on other people.'

Mariam knew that her life was in danger, that she may lose a lot of blood after delivery and if she was in the hospital then midwives could do something; even give her blood. Mariam wanted to go but she did not have the money. The day labour started, Mariam’s husband had gone into town. Her sister and aunt came.

'I told them I wanted to go to hospital for the delivery. Auntie told me that we can only go to the dispensary, and if there is a serious complication, they will get transport, so I can go to the hospital. I sat on the bicycle, which was pulled by one of my older children.'

Labour pains had been very strong and she delivered immediately on arrival to the dispensary. When the midwives asked why she did not go to the hospital, ‘I told them my husband was away’.

### Narrative III: Ashura – passive and moving with the wind

Ashura was 17 years old; she and her husband were living in her parents-in-law’s compound, together with other relatives. When Ashura was 7 months pregnant, the antenatal care midwife told her that she had insufficient blood (anaemia); therefore, she was advised to go to hospital for further assessment and treatment.

'But I did not have any problems, except for some weakness. I have heard that some women are held at the hospital in a ward with less service than the normal ward for failing to pay bills. They have named the ward ‘Mortuary’ as a woman and her relatives who cannot pay are like dead bodies.'

Ashura’s husband was away, so she informed her mother-in-law. Her mother-in-law said that there are problems for which one must go to hospital immediately, such as high blood pressure, being too sick or a small passage. ‘My mother-in-law said that she once had the same problem and was told to go to the hospital but did not go. She used medicines for blood and became better’. Her mother-in-law promised to discuss the issue with her son when he returned. In the meantime, the mother-in-law bought medicines for her to use.

Ashura started using the medicines. Her husband came back after a few days and said he had some money; it was 60,000 Tanzanian shillings (equivalent to 30 USD). Ashura told him that the money was not enough to pay for transport, hospital bills and other living expenses at the hospital. ‘But, I was not sure if that is all the money he had, as he was keeping all the money and he did not tell me how much he had saved’.

Ashura was worried that they could be held at the hospital if her husband and parents-in-law failed to pay the bills. Being held at the hospital for a long time would increase the bills and she would be separated from her first baby for a long time. Ashura also felt that other people could look down upon her and her husband.

At 9 months, the midwife at the dispensary insisted on hospital delivery since her ‘blood was too low’ to deliver at the dispensary. Ashura felt that her body was weak but she had no dizziness or blackouts. She continued using the medicines given by her mother-in-law.

The same week she felt labour pains. ‘I went to the dispensary, and I delivered safely. I didn’t pour a lot of blood, and I was not confused and nothing happened’. After the delivery the midwife asked her about the referral. ‘I told her I didn’t have enough money to go to the hospital’.

## Discussion

The study reveals that when women decline maternal referral advice given at a dispensary, the decision-making process usually involves interactions with other family members and depends on the prevailing family and economic situation at the time. This illustrates the pregnant woman’s dependence on informal networks and her limited individual ability to act autonomously. However, some pregnant women were able to make their own decision about whether to accept or decline the referral advice given by health workers, while other women remained passive and dependent on others’ decisions. As opposed to the common hegemonic masculinity discourse where the husband makes all of the decisions alone [22,23], in this study some women were viewed to be knowledgeable on pregnancy matters and were able to make decisions on referrals.

In agreement with previous studies, these results showed that pregnant women and other actors have ways of evaluating the risks and complications needing referral which differ from the biomedical perspective of the health workers [11,13]. The actors’ perception was that a pregnant woman who is advised to go and deliver in the hospital should go only when she has failed to deliver at the dispensary, despite being aware of the increased costs and the risk that the mother may die or have a permanent disability. Looking out for complications in a high-risk pregnancy may be possible in areas where facilities are well equipped and health workers are available to deal with any complications that should arise, and if the transport and infrastructure are good. In such circumstances, the woman can be transferred to the hospital immediately for treatment. This is not the case in most low-income countries, including the current study setting [24,25].

The concepts of power, economic, affection and symbolic dimensions of gender discussed by Connell [18], as well as the positive and negative aspects of social capital [14,17], are used to explore interactions within and among pregnant women and other actors in declining the professional advice for maternal referral for complications in pregnancy.

### Power dimension

The power dimension of gender often demonstrates the subordination of individuals or groups [18], and is reflected and illustrated at individual and family levels in the current study. At an individual level, however, some pregnant women in this study took the decision to decline the referral and made sure that their wishes were fulfilled by significant others in the family. These pregnant women represent a group of women who are assertive and can make independent decisions regardless of what others around them are thinking, and are not seen as powerless. If economically empowered and given the right information, most women can make a positive impact in their own lives and the lives of other family members. Conversely, the family institution, especially the mothers and mothers-in-law, were often more powerful in making decisions for some pregnant women who had intended to go for the referral, and these women had to comply with the wishes of others. To these women, the social capital of respect and adhering to social norms [14] were of great importance given their dependence on future support in the case of problems or complications during pregnancy and delivery. However, as a result it may have negative effects on the health of the pregnant woman and/or the baby, as in this study some women were misinformed about the medical procedures provided in the hospitals.

In general, the information from the midwives advising on referral was not given much credit by the pregnant women’s mothers and mothers-in-law. In many cases, the referred women’s mothers and mothers-in-law had the power to evaluate the referral advice and make their own risk calculation. They were viewed by other actors as being knowledgeable on matters related to pregnancy and childbirth. This illustrates not only the power [18] that these experienced women have in deciding issues related to pregnancy and childbirth, but also the great trust [14] given to the decisions they make. On other hand, it contradicts the common discourse and gender stereotype that men have decision-making power in all family matters, including issues related to maternal health [23]. Pregnant women, husbands and fathers reported to the older women and asked for their opinion in all referrals, often disregarding the referral advice given by health workers. Mothers and mothers-in-law had the power to decide whether professional advice would be taken, and when and where to go. The position of the mothers and mothers-in-law was especially visible if they were traditional birth attendants, as they were seen to be more knowledgeable and experienced. The study showed they were acting as gatekeepers for the pregnant women around issues of referral. These findings have implications when planning reproductive healthcare interventions and indicate that these experienced women are an important group to be targeted. Using the concept of trust, an important aspect of social capital, investing in these women, in whom the community displays great trust, may be an important step in strengthening referral systems from the community perspective.

Some pregnant women demonstrated opinions on the referral advice, either accepting or declining the advice. The power dimensions at different levels, i.e. individuals, pregnant women, their families and significant others, came into conflict when some pregnant women made their own decisions and took a different stand to accept or decline the referral. Despite these pregnant women having their own opinions, there were economic, affection and symbolic dimensions of gender relations in the family and community institutions which hindered them from pursuing their course. The relation between these dimensions at that specific time and institution were used to reach a decision.

### Economic dimension

The economic dimension explains the external financial needs in case of events and the distribution of available resources [18]. Financial capability played a part in the interactions between pregnant women and their husbands during the referral advice. Pregnant women found themselves assessing the financial ability of their husbands and other actors before making a decision about the referral advice. Figuring out how much money their husbands, parents, parents-in-law and other actors have puts pregnant women in the difficult situation of not knowing what will be decided. Independent of their decisions, pregnant women tended to be economically dependent on their husbands and other people around them to be able to go for referral. Pregnant women and their mothers and mothers-in-law all depended on men to provide economic support during referrals. However, the mothers and mothers-in-law had more power in decision making and, therefore, most of the pregnant women had to follow their decisions to guarantee emotional, social and economic support in future. This illustrates another social capital concept [14] known as ‘reciprocity’, where a woman had to adhere to social norms and follow orders to receive the support she might need in future from the same people.

Engaging in small-scale farming and petty business in this rural community was seen as the income generator for the family which enabled the women to go for the referral, but this was gendered towards the husbands, fathers and fathers-in-law. The women, including the mothers and mothers-in-law, were not seen as being instrumental in making money to contribute financially to the referral.

The economic dimension extended to some pregnant women being taken to their parents or parents-in-law for care while their husbands were working in other areas to find money for the referral. Although this may be seen as a symbolic dimension of care for the children, it is also an economic dimension to earn money which may be needed for the referral. The absence of the husband when the woman is in labour and referral is imminent, and when the woman needs his emotional support, may be surpassed by the economic pressures experienced by the family.

All pregnant women and actors were sure that they would be able to obtain the money and transport to go to the hospital in case of an emergency and if the life of the woman was in immediate danger. Mselle et al., studying women with obstetric fistulae in Tanzania, reported that decision to seek care in health facilities was reached when a complication had occurred [26]. The finding that more people are engaged when a woman is in labour, and especially when labour takes longer than normal, means that not only is the economic dimension important, but also the affection and symbolic dimensions of values of saving a life become important.

### Affection/emotional dimension

The emotional dimension involves the capacity to manage one’s feelings and limitations, and may be in conflict with the economic and symbolic dimensions [18]. The pregnant women’s intention to go for referral was in direct conflict with the need to take care of their children and families at home. Being a mother and wife and fulfilling the responsibilities pertaining to these two roles was important but challenging to the pregnant women if they had to accept the referral. For the pregnant women, being ‘a good mother’ or ‘good motherhood’ is directly attached to fulfilling motherhood roles in taking care of their children. The pregnant women were not ready to surrender their motherhood role, even temporarily, to go for the referral that would ensure their own well-being and that of the unborn child and their family in the future. Although the love and intention to take care of the children shows their intimate relationship with their children and therefore affection to their children, it can also be interpreted as the women needing to maintain their symbolic respectability in the community. Despite the efforts of the mothers and mothers-in-law to ensure that there was someone at home to take care of the children, pregnant women in this study felt that their positions as carers of their own children could not be completely fulfilled by other carers.

Fear of surgery if the woman went to hospital was explained in relation to the potential inability to carry out domestic chores after the operation, which requires longer recovery compared to a normal and spontaneous delivery. This may make the women feel physically unable to take care of their children, which may be perceived as not displaying love and intimacy.

### Symbolic dimension

The symbolic dimension reveals the sociocultural relations [18] that may, in practice, hinder a pregnant woman’s ability to access the appropriate services. All actors embraced the perception that men, as husbands, fathers and fathers-in-law, were respected owing to their economic capability. Provision of money for feeding the family and being able to pay for the referral costs if the woman needed to go to hospital were symbols of manhood, thus maintaining their reputations among themselves, the family and the village. Being unable to pay for hospital bills was seen as damaging the status and respect, not only of the husband but of the whole family.

The status of husbands in the family and village due to their economic contribution was challenged by the status of mothers and mothers-in-law having knowledge on pregnancy and childbirth issues. They maintained their status during the referral advice. Because of their knowledge, mothers and mothers-in-law were relied on to make decisions about the referral advice and when to go to the hospital, while husbands maintained their status by being ready to bear costs. In declining referral and deciding to deliver in the dispensary, the respect of the mothers and mothers-in-law and their symbolic power increases when the women deliver without complications, and may decrease if the women end up with severe complications. Conversely, the symbolic power for the health workers is continuously damaged, especially if declining a referral resulted into a positive outcome for the mother and the unborn child. This sets a precedent for future decisions, where there is an increased chance of referrals being declined by these women or their family members, friends, acquaintances and neighbours.

### Limitations of the study

This study focuses only on pregnant women who were advised at a dispensary to deliver in a hospital, but did not take up the advice. This is a unique group, as all of these women went back to the dispensary and delivered without complications to either the mother or the baby. More insight might have been gained if we had included women who reached the hospital or gave birth at home, or relatives of women who had died because they did not follow the referral advice.

The study did not explore in detail the interactions between the health workers and the pregnant women and how these influenced decisions regarding referral. This was due to fact that we included women who had declined referral advice. We cannot ignore the possibility of social desirability bias in the responses from the respondents. However, as the interviews were conducted in a non-judgemental way, we felt that the respondents answered openly and were not hiding any information.

The findings of this study cannot be generalized to all pregnant women in Tanzania, but can be used to show how pregnant women who are given referral advice may not comply because of other circumstances such as socio-economic and family pressures.

### Strengths and trustworthiness of the study

To ensure credibility, we have described the data using narrative research with systematic analyses of the data, staying close to the transcripts and formulating three stories with anonymized characters to illustrate the various ways in which advice from health workers was handled. Repeated reflection during the analyses was felt to be crucial; this, and the diversity of the research team and their different experiences, added to the credibility of the findings. The context and respondents were described to approve transferability, giving the reader the possibility to relate the findings to other settings. To ensure dependability, direct quotations from the respondents have been added in the narratives to illustrate their views on the advice. Furthermore, the study was conducted by the same group of researchers throughout the study to maintain consistency. Using Kiswahili for data collection ensured a smooth interaction with the respondents and the researchers and meant that the respondents were able to express their views and communicate freely. This contributed to the confirmability of the study. Furthermore, the researchers involved in the data collection and analysis had extensive knowledge and understanding of maternal health in this specific Tanzanian context.

## Conclusion

The study reveals that declining the maternal referral advice given at a dispensary involves interactions with family members and depends on the prevailing family and economic situation at the time. Although the decision to accept or decline the referral advice was greatly influenced by family members, the individual pregnant woman’s position in relation to the referral advice played a specific role in the decision-making process. Other women identified in the support network had great decision-making power, which contradicts the common discourse and gender stereotype that men have decision-making power in all family matters, including issues related to maternal health. If women are socially and economically empowered, both those of reproductive age and older women, this could positively influence decision making during maternal referrals, thus reducing the first delay in seeking care. Furthermore, educational interventions with community members are needed to improve the awareness of referral and its acceptance. More studies are needed to explore and understand the complex interrelations that may influence maternal referral acceptance.

## Paper context

In lower- and middle-income countries most women attending antenatal care do not follow referral advice despite having high-risk pregnancies or complications. This study in Tanzania explores how antenatal maternal referral advice is handled in making the decision not to follow the advice. The study revealed that women’s failure to follow referral advice is embedded within their position, lack of independence and low autonomy. There is a need to empower women to increase their acceptance of referral.
